# Altering phosphorylation in cancer through PP2A modifiers

**DOI:** 10.1186/s12935-023-03193-1

**Published:** 2024-01-06

**Authors:** Hannah Johnson, Satya Narayan, Arun K. Sharma

**Affiliations:** 1 Department of Pharmacology, Penn State Cancer Institute, The Pennsylvania State University College of Medicine, Hershey, PA 17033 USA; 2 Department of Anatomy and Cell Biology, University of Florida, Gainesville, FL 32610 USA

**Keywords:** PP2A, Activators, Inhibitors, Cancer, Therapeutics

## Abstract

Protein phosphatase 2A (PP2A) is a serine/threonine phosphatase integral to the regulation of many cellular processes. Due to the deregulation of PP2A in cancer, many of these processes are turned toward promoting tumor progression. Considerable research has been undertaken to discover molecules capable of modulating PP2A activity in cancer. Because PP2A is capable of immense substrate specificity across many cellular processes, the therapeutic targeting of PP2A in cancer can be completed through either enzyme inhibitors or activators. PP2A modulators likewise tend to be effective in drug-resistant cancers and work synergistically with other known cancer therapeutics. In this review, we will discuss the patterns of PP2A deregulation in cancer, and its known downstream signaling pathways important for cancer regulation, along with many activators and inhibitors of PP2A known to inhibit cancer progression.

## Background

Phosphorylation in mammalian cells is largely regulated by a combination of kinases and protein phosphatases [1]. Alterations in the activity of these processes can result in a variety of diseases, including cancer, diabetes, and neurodegenerative diseases [1–3]. Phosphatases can be classified into three main categories: protein tyrosine phosphatases (PTPs), protein serine/threonine phosphatases (PSPs), and dual-specificity phosphatases (DSPs) [4]. The PSPs are then sub-categorized into three groups: phosphoprotein phosphatases (PPPs), Mg^2+^/Mn^2+^-dependent protein phosphatases (PPMs), and aspartate-based phosphatases. The PPPs contain enzymes such as PP1, PP2A, PP2B (activated by calcium), PP4, PP5, PP6, and PP7 [1]. As serine/threonine phosphorylation in mammalian cells accounts for over 98% of protein phosphorylation, the major inclination of these enzymes to form oligomeric complexes is fundamental, with only a small number of genes encoding for PSPs. By this nature, protein phosphatase 2A (PP2A) is able to form into multiple combinations of the heterotrimeric holoenzyme, which allows for its association with 50–70% of all serine/threonine phosphatase activity [4–6]. Due to its role as a tumor suppressor, there is a tendency for dysregulation of PP2A in cancer which can result in decreased regulation of tumorigenic pathways, and subsequently trigger aberrant cell progression. This problem has led to considerable research focused on targeting PP2A in the treatment of cancer through either its activation or inhibition. One of the advantages for utilizing a PP2A modulator, instead of a kinase inhibitor, lies in the substrate specificity of PP2A due to its large diversity of holoenzyme combinations. The regulatory subunits utilized in the formation of each holoenzyme restrict the substrate specificity of PP2A such that therapeutics targeting a specific PP2A holoenzyme have the potential for less off-target effects than observed with kinase inhibitors [4, 7].

PP2A is classified as a heterotrimeric holoenzyme, which is made up of three subunits: the scaffolding A subunit (PP2Aa or PP2A-A), the catalytic C subunit (PP2Ac or PP2A-C), and the regulatory B subunit (PP2Ab or PP2A-B). Both PP2Aa and PP2Ac have two isoforms (α and β) with the α isoform being predominantly expressed. The B subunit has 15 genes, which can be spliced to encode for 26 regulatory subunits overall. Each of the regulatory subunits can be clustered into four families: B (B55/PR55), Bʹ (B56/PR61), B″ (PR48/PR72/PR130), and B‴ (PR93/PR110) with a sequence homology only shared among the subunits within each family [1, 4, 8]. The downstream signaling pathways affected by PP2A activation are largely dependent on which regulatory subunit is used in the formation of the holoenzyme. During PP2A biogenesis the scaffolding and catalytic subunits will bind together to form an AC dimer; however, before this occurs the activation site within PP2Ac needs to be activated. Free PP2Ac in the cell will be ubiquitin tagged for degradation by ubiquitin E3 ligase Midline 1 (MID1) [9]. In order to prevent degradation, free PP2Ac is bound by α4 to produce a stable pool of latent PP2Ac, which can then be used in the biogenesis of PP2A once activated [10]. During activation the phosphotyrosyl phosphatase activator or PP2A-specific phosphatase activator (PTPA) induces a conformational change to the catalytic subunit before PP2Ac binds to PP2Aa and forms the PP2A-AC dimer [11]. Due to the large range of PP2A holoenzyme forms, some differences in the binding of the regulatory subunit to the AC dimer are needed. Previously, the substrate specificity of the regulatory subunits were thought to be connected to structural differences between subunits rather than a specific sequence motif, due to the large degree of conformational flexibility observed with the scaffolding subunit [4, 12]. More recently, researchers have identified short linear motifs (SLiMs) within intrinsically disordered regions (IDRs) for the B55 and B56 regulatory subunits, suggesting that PP2A substrate specificity follows a similar binding mechanism to other PPP molecules [13–15]. Further distinctions made between PP2A holoenzyme formations can be found in the post-translational modifications at the carboxy-terminal tail of PP2Ac [16]. Primarily, the phosphorylation of T304 and the carboxymethylation of L309 are the main post-translational modifications on PP2Ac [17]. The B (B55/PR55) subunits need the methylation at L309 to form a stable holoenzyme using leucine carboxyl methyltransferase 1 (LCMT-1) to methylate the carboxy terminus of PP2Ac which can then be demethylated by protein phosphatase methyltransferase 1 (PME-1). Demethylation by PME-1 can change or decrease this holoenzyme formation by destabilizing the binding of the B (B55/PR55) subunit [4, 18]. Previously, it was thought that phosphorylation at Y307 on PP2Ac was also related to cancer progression and PP2A inhibition; however, recent validation experiments on the p-Y703 antibody have shown a lack of specificity towards this residue. These recent findings, along with the lack of mass spectrometry data of p-Y307, have led to revaluation of the studies utilizing this antibody [19]. The degree to which each PP2A holoenzyme requires specific conformation or post-translational modifications is still being studied. Modifications to these specifications have been linked to cancer, leading to PP2A modifiers being developed to address this loss of serine/threonine phosphatase activity.

**Fig. 1:**
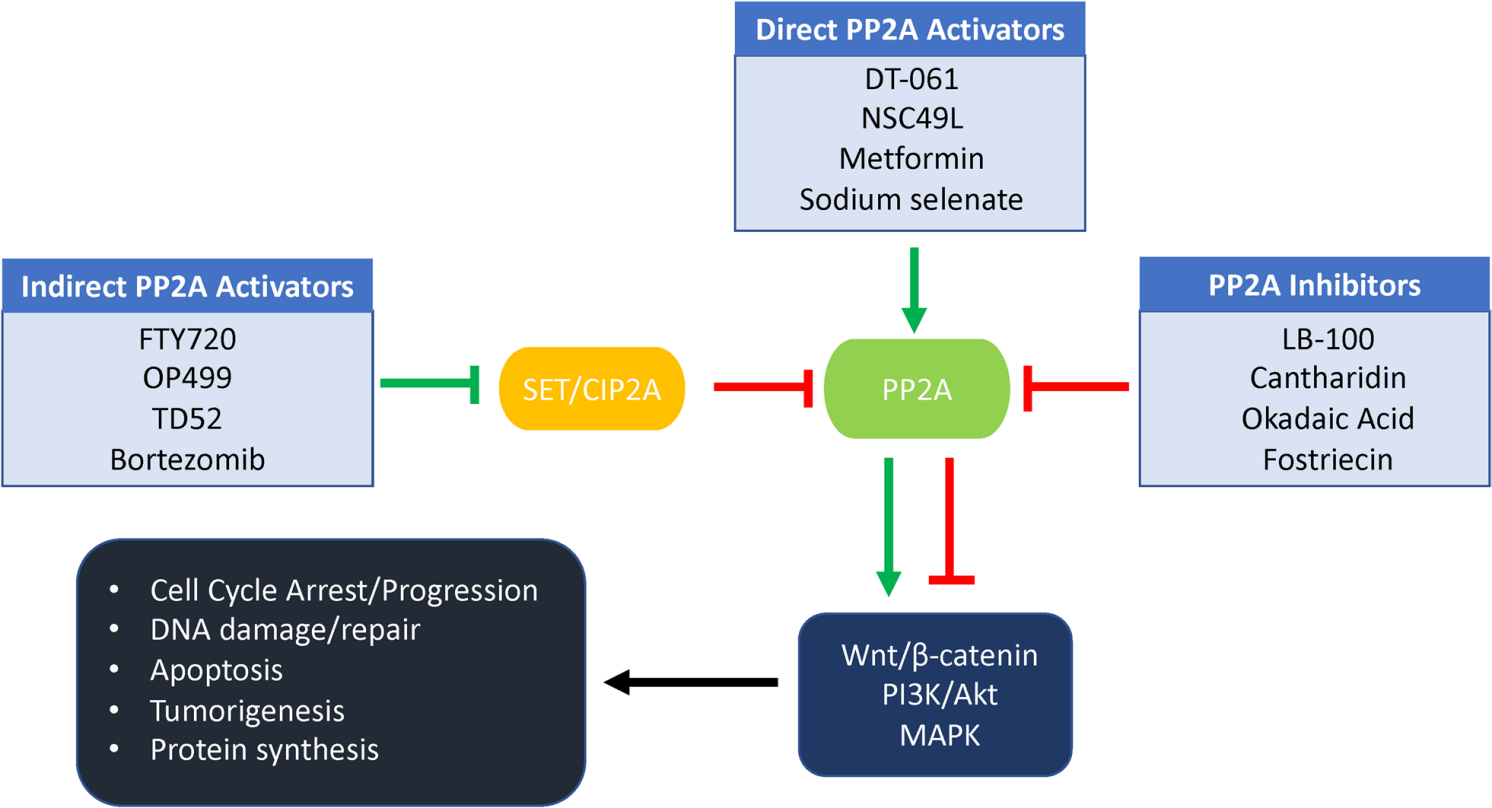
Overview of the downstream effects of PP2A therapeutics. Indirect PP2A activators inhibit the endogenous protein inhibitors of PP2A resulting in increased PP2A activation and increased regulation of downstream pathways, such as Wnt/β-catenin, PI3K/Akt, and MAPK. Direct PP2A activators can bind directly to the protein and promote its activation leading to increased regulation of pathways involved in tumor progression. PP2A inhibitors can bind to the protein and prevent dephosphorylation of its substrates leading to increased cell cycle progression and eventually cell death due to DNA damaged cancer cells

In this review, we will be considering the effect of therapeutic PP2A modifiers in cancer. Both the drug-induced activation and inhibition of PP2A have been found to inhibit cancer, which may seem like a contradiction; however, the large diversity of PP2A holoenzymes can explain this prospect. The specific PP2A holoenzyme activated by these modifiers can lead to different downstream signaling pathways normally regulated by PP2A. In addition, in cancer, depending on the regulatory subunit, PP2A activity may inhibit cancer progression (Fig. 1) [20].

## PP2A deregulation in cancer

PP2A activation is largely involved in many signaling pathways essential to the hallmarks of cancer. As such, PP2A characteristically functions as a tumor suppressor and is known for being frequently inactivated in many cancer types [21]. For instance, PP2A has been extensively observed to be deregulated in colorectal, breast, oral squamous cell carcinoma, and acute myeloid leukemia (AML) cancer patients [22–25]. Moreover, a PP2A inhibitor, okadaic acid (OA), was applied onto the skin of mice where they subsequently developed tumors, indicating that a loss of PP2A activity can assist in cancer development [26, 27]. The deactivation of PP2A in cancer is typically caused by mutations, post-translational modifications, or the increased expression of endogenous PP2A inhibitors, such as Suvar/Enhancer of zeste/Trithorax (SET) or cancerous inhibitor of PP2A (CIP2A) [8].

For instance, an *in-silico* analysis of breast cancer patients found that approximately 59.6% of the basal breast tumors contained deregulated PP2A. About 46.7% of these patients had either low expression of one of the PP2A subunits or high expression of the inhibitory regulatory subunits, while 8.6% of these patients reported high expression of CIP2A or SET [23]. These findings provide a good illustration of the mechanistic variability in PP2A dysregulation in tumors. Additionally, the analysis of 21 colon cancer (CRC) patients showed a decrease in PP2A activity, which was likely caused by the observed overexpression of both SET and CIP2A in CRC as well as the downregulation of the regulatory subunits, *PPP2R2A* and *PPP2R5E* [22]. This deregulation of PP2A has been found in the patients of many different cancers, showing a common therapeutic target across cancer types. Mutations in *PPP2R1B* and *PPP2R1A*, the genes for the α and β isoforms of the PP2Aa subunit, have been found in breast and lung carcinomas as well as melanomas [28, 29]. *PPP2R1A* mutations have been observed to occur at high frequency in endometrial carcinoma and at low frequency in ovarian cancer [30, 31]. Moreover, point mutations or deletions of *PPP2R1B* were found to disrupt the binding of PP2Ac with PP2Aaβ in CRC patient tissues contributing to the deregulation of PP2A in CRC, though the frequency of *PPP2R1B* mutations in cancers is low [28, 32]. Furthermore, deletions of *PPP2R2A,* the gene for PP2A-B55α, have been in both breast and prostate cancers [33–35]. Studies also showed that 70% of cancer patients have a heterozygous deletion or a missense mutation in *PPP2R4*, the gene for PTPA, the cellular PP2A activator. The mutated PTPA was observed to have decreased binding with the PP2Ac subunit and a decline in its ability to activate PP2A [36]. These instances of PP2A dysfunction in cancer patients lead to the deregulation of many anti-cancerous downstream pathways; whereby, the activation of PP2A can restore normal function in cells allowing for cytotoxicity in cancer cells.

## PP2A downstream signaling targets in cancer

PP2A directly and indirectly affects many signaling pathways integral for either cancer progression or cancer inhibition. These downstream signaling pathways may include the regulation of apoptosis, cell proliferation, cell cycle progression, and tumorigenesis among many others [4, 8]. The specific downstream pathway regulated by PP2A activity is largely reliant on the regulatory subunit bound to the holoenzyme, meaning that a great deal of interplay occurs with a PP2A holoenzyme in multiple signaling pathways [37]. As such, some of the major signaling pathways regulated by PP2A are the PI3K-AKT, Wnt, mTOR, and MAPK, accounting for the association between PP2A dysfunction and cancer progression [38].

**Fig. 2:**
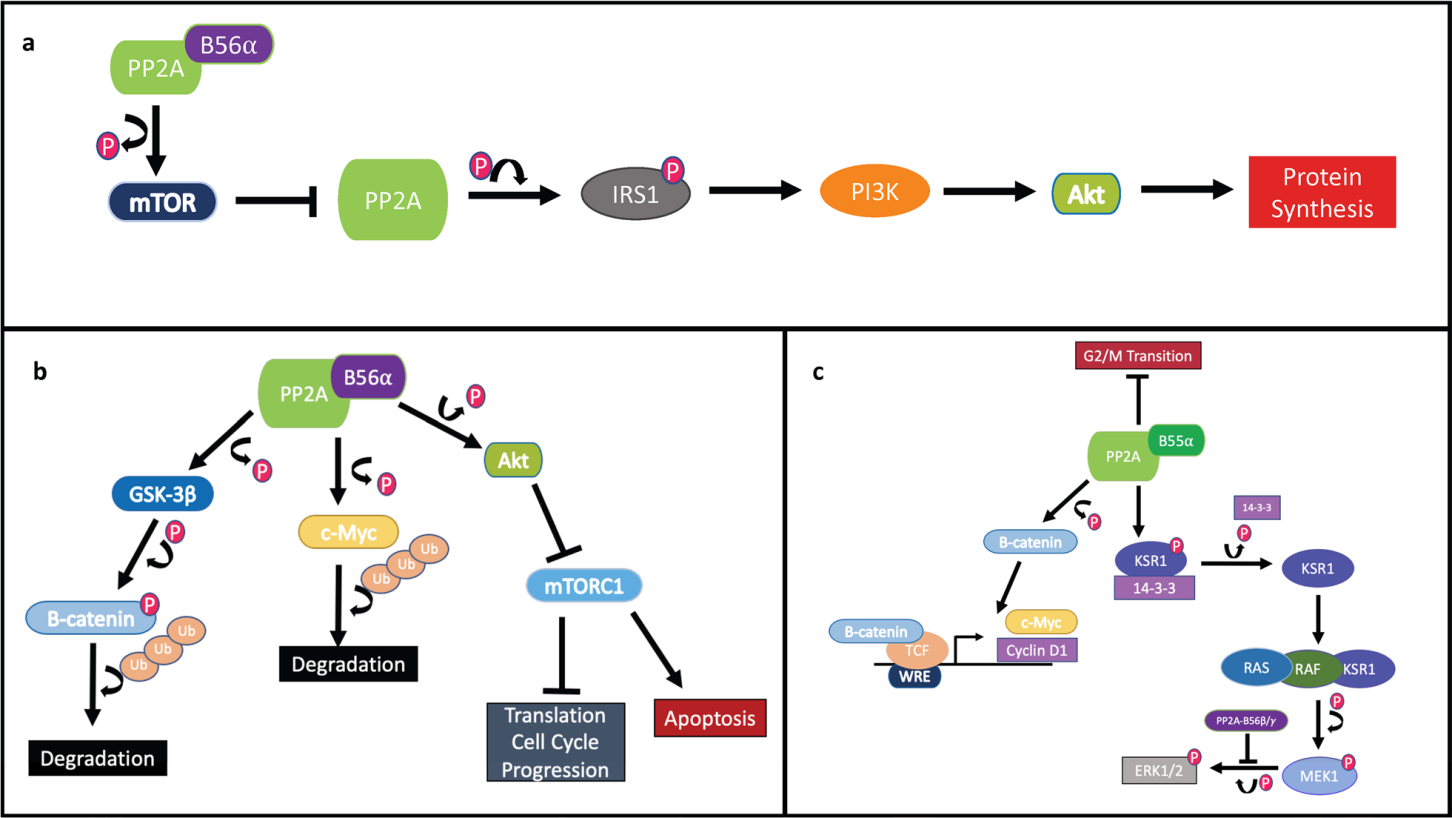
**A** PP2A-B56⍺ dephosphorylates mTOR downregulating its activity. mTOR, alternatively, can inhibit PP2A leading to dephosphorylation of IRS1. p-IRS1 activates PI3K/Akt signaling leading to increased protein synthesis. **B** Some of the PP2A-B56⍺ downstream signaling. 56⍺ dephosphorylates GSK-3β activating it and allowing GSK-3β to phosphorylate β-catenin resulting in ubiquitin tagging for degradation in the ubiquitin–proteasome pathway. B56⍺ also dephosphorylates c-Myc resulting in it being tagged by ubiquitin for degradation. PP2A-B56⍺ can also dephosphorylate Akt, inhibiting mTORC1, and resulting in the inhibition of translation and the cell cycle progression. **C** PP2A-B55⍺, on the other hand, can dephosphorylate β-catenin allowing it to activate the Wnt responsive genes (WRE) and transcribe the Cyclin D1 and c-Myc. B55⍺ dephosphorylates KSR1 causing its disassociation from the 14-3-3 complex and binding to the RAS-RAF complex, which phosphorylates MEK1. Activated MEK1 then phosphorylates ERK1/2 activating it for downstream regulation. PP2A-B56β/γ is the subunit known to dephosphorylate ERK1/2 reversing these activations

The Wnt signaling pathway is fundamentally associated with the cell cycle, as it initiates cell cycle progression by inducing the transcription of the cell division promoters. Wnt signaling is both positively and negatively regulated by PP2A due to its role in β-catenin regulation. For instance, PP2A-B56α is able to promote β-catenin degradation by dephosphorylating GSK-3β. On the other hand, PP2A-B55α dephosphorylates β-catenin resulting in its accumulation in the nucleus where β-catenin will then bind to the T-cell factor (TCF) transcription factor allowing for the promotion of Wnt-responsive genes, such as *cyclin D1* and *c-Myc* [4, 37]. PP2A-B56α is also involved in the ubiquitin-tagged degradation of c-Myc through the dephosphorylation of GSK-3β and c-Myc. The stabilized form of c-Myc is phosphorylated at S62 which triggers its phosphorylation at T58 by PP2A-activated GSK-3β. Once both the S62 and T58 residues are phosphorylated on c-Myc, PP2A-B56 will dephosphorylate S62 leading to c-Myc being ubiquitin-tagged for degradation (Fig. 2B, C) [37]. PP2A has also been shown to control the G1/S transition with B56γ3, preventing the G2 to M transition with B55α, and positively regulating cells mitotic exit with B56δ [38–41]. This signaling pathway strongly exhibits the contrary roles PP2A can play in signaling pathways all of which are highly dependent on the regulatory subunit bound to the holoenzyme.

The mechanistic target of rapamycin (mTOR) signaling pathway, which is highly associated with the PI3K/AKT cascade, is implicated in cell growth, survival, and protein synthesis [42]. PP2A is mainly associated as a negative regulator for the mTOR signaling pathway. PP2A-B56α and PP2A-B55α are known for dephosphorylating AKT1 at T308 and, under specific conditions, S473, leading to its inactivation and the inactivation of mTORC1 (Fig. 2B) [43–45]. Subsequently, as S6K and 4E-BP1 are the main substrates of mTORC1, translation is inhibited, and as the phosphorylated AKT1 assists in cell cycle progression and the suppression of pro-apoptotic proteins, there are many downstream effects of PP2A-induced inactivation of AKT1 [46, 47]. PP2A-B56α has also been found to dephosphorylate and inactivate mTOR, while PP2A-B56γ directly dephosphorylates p70S6K halting further downstream signaling of the mTOR signaling pathway [4, 48, 49]. Conversely, mTOR can inhibit PP2A directly, as well as allowing for the regulation of insulin receptor substrate 1 (IRS1), the upstream protein of AKT that is needed for the induction of insulin receptor signaling to PI3K [50]. The PP2A-induced dephosphorylation of IRS1 inhibits the activation of PI3K, while the mTOR-induced inhibition of PP2A results in the phosphorylated IRS1-mediated PI3K/AKT signaling, and thus increases protein synthesis (Fig. 2A) [4, 50]. As PP2A works to negatively regulate the mTOR signaling pathway, there are many feedback loops to assist in the further control of this pathway, though it is still frequently deregulated in cancer cells which eventually leads to aberrant cell progression, diminished apoptosis, and increased protein synthesis [42].

The mitogen activated protein kinase (MAPK) signaling pathway mainly contributes towards the functions of proliferation and apoptosis [51]. Consequently, it plays a large role in tumorigenesis. The four cascade families encompassed in MAPK signaling include, ERK1/2, JNK, p38, and ERK5. The ERK5, JNK, and p38 cascades typically signal an induction to apoptosis as a response to stress, while the ERK1/2 stimulates cellular proliferation [4]. As ERK1/2 can promote c-Myc stabilization by S62 phosphorylation and initiate Jun and Fos transcription factor activation for cell cycle regulation, its regulation is an important target in cancer inhibition [52, 53]. The function of PP2A in the MAPK signaling pathway then understandably initiates both positive and negative regulation. PP2A-B56β/B56γ was found to dephosphorylate ERK1/2, resulting in its inactivation and the negative regulation of this pathway [20]. On the other hand, PP2A-B55α dephosphorylates kinase suppressor of Ras 1 (KSR1) and Raf-1, allowing for disassociation from the 14-3-3 complex and activation of MEK1 that ultimately results in the positive regulation of MAPK signaling (Fig. 2C) [54]. Many processes are linked to the Wnt, mTOR, and MAPK signaling pathways and many parts of these processes intersect with each other; however, the contribution given by PP2A during normal function gives rise to its importance as a target for therapeutics in cancer treatment [8, 51].

## PP2A activators

As PP2A is a significant regulator for cellular function, its cancer-induced dysregulation presents an optimal target for therapeutic modulation. Therapeutics targeting the activation of PP2A in cancer function by rescuing the normal positive PP2A regulation of tumor suppressor signaling pathways [55]. Because PP2A is dysregulated through a few different pathways, there are two classes of PP2A activators, direct and indirect activators [56]. The direct PP2A activators work by directly binding to the holoenzyme to induce its activity [57]. On the other hand, the indirect PP2A activators function by inhibiting the endogenous inhibitors for PP2A, such as CIP2A or SET. Both classes will positively influence the activation of PP2A; however, there will be differences in downstream signaling regulation dependent upon which PP2A holoenzyme combination was therapeutically activated [8, 57].

### Direct PP2A activators

**Fig. 3:**
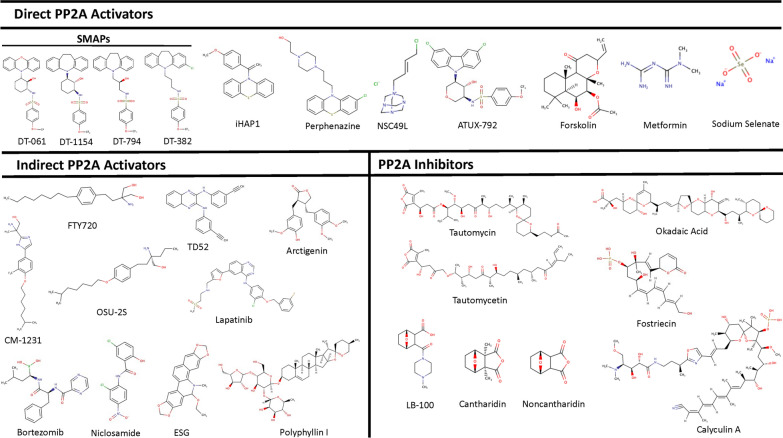
Chemical structures of the PP2A modulators. MarvinSketch was used for drawing chemical structures, Marvin 17.21.0, Chemaxon (https://www.chemaxon.com)

The direct PP2A activators will bind to the holoenzyme to induce activation; however, the downstream signaling regulated from this activation will change contingent on the regulatory subunit involved (Table 1) [57]. Therapeutics in this class will likely have more substrate specificity to directly target neoplastic drivers relevant to cancer progression. The method by which these therapeutics directly bind to PP2A is still being discovered; however, the current therapeutics largely indicate that it highly depends on the compound itself (Fig. 3). As PP2A therapeutic targeting in cancer is considered a prevalent avenue of potentially significant cancer treatment, more research into identifying and designing novel PP2A modulators is continually occurring.

**Table 1:** Direct PP2A activators

Compounds	Cancer types	Mechanisms	References
SMAPs: DT-061 DT-1154 DT-794 DT-382	Burkitt’s Lymphoma	Myc degradation via dephosphorylation at Ser62	[55, 62]
	KRAS mutant NSCLC		
	Triple Negative Breast Cancer		
	Multidrug resistant chronic lymphocytic leukemia (CLL)	Apoptosis activation through inducing mitochrondrial permeability transition pores (mPTPs)	[63]
	Glioblastoma	Blood–Brain Barrier Permeable	[134]
	TKI-resistant advanced lung adenocarcinoma	Downregulation of PI3K and MAPK pathways	[61]
	Neuroblastoma	Decreased MYCN	[68]
iHAP1	T-ALL	Dephosphorylates MYBL2 Inhibits tubulin polymerization	[64, 69]
Perphenazine	T-ALL	Dephosphorylates c-Myc, AKT1, p70S6K, ERK, and BAD	[58–60, 69]
NSC49L	Colorectal Cancer	Decreased AKT11, mTOR, 4E-BP1, p21	[70]
	5-FU resistant Colorectal Cancer		
ATUX-792	Neuroblastoma	Decreased MYCN (prognostic factor for neuroblastoma)	[68]
ATUX-3364	Hepatoblastoma	Decreased cell cycle progression, motility, and stemness, decreased mRNA expression of OCT4, NANOG, SOX2	[67]
ATUX-8385			
Forskolin	Colorectal cancer	Dephosphorylates PP2Ac	[135]
	Acute myeloid leukemia	Caspase-dependent apoptosis, decreased phosphorylated Akt and ERK1/2	[24]
Metformin	Breast Cancer	Decreased phosphorylated Bax, c-Myc, and AKT1	[71, 72]
	Lung Cancer		
	Prostate Cancer		
Sodium selenate	Castration-resistant prostate cancer	Decreased angiogenesis	[73–75]
	Hormone-refractory prostate cancer

The most well-known class of molecules identified for inducing PP2A activation are phenothiazines, which are tricyclic neurotropic drugs [58]. Though phenothiazines are typically used as antipsychotics, they have been recognized to induce anti-cancerous effects [58, 59]. In recent studies, it has been shown that phenothiazines can initiate anti-cancerous effects through the activation of PP2A [59, 60]. However, as phenothiazines tend to be nonselective, there are dose-limiting extrapyramidal side-effects through CNS (central nervous system) receptor binding [58]. To address these side-effects, a series of phenothiazines analogues were synthesized to prevent this nonselective binding while still maintaining their anti-cancerous features. These analogues were assessed for both anti-cancer activity as well as their abilities in activating PP2A [58]. From this study, a series of molecules were identified known as small molecule activators of PP2A (SMAPs), where SMAPs were observed to bind via PP2A-Aα to induce the conformational change needed to induce activation of PP2A [55]. More recently, DT-061, a SMAP, has undergone further study into the mechanism behind its binding to PP2A [55, 61]. It was discovered that DT-061 interacts with the three PP2A subunits forming a binding pocket where moieties of the compound can interact with various residues of the three PP2A subunits. For instance, the phenoxazine moiety of DT-061 was found to interact with the Y307 residue of the catalytic subunit sticking the carboxy terminal tail of PP2Ac between the scaffolding and regulatory subunits which implied better stability for this heterotrimeric complex [57]. The binding of DT-061 prevents the destabilization of the PP2A-B56α holoenzyme, allowing for the identification of the known downstream substrate specificity of DT-061, such as the dephosphorylation of c-Myc [57, 62]. The SMAPs have been found to be effective PP2A therapeutics for a variety of cancers both in vitro and in vivo, including overcoming drug resistant cancers. For instance, DT-061 has been observed to overcome the drug resistance of venetoclax (VEN) in chronic lymphoblastic leukemia (CLL) through the activation of apoptosis. As VEN-resistance is due to the inhibition of Bax/Bak induced apoptosis, DT-061 can induce mitochondrial permeability transition pores (mPTPs) and activation of apoptosis without the Bax/Bak pathway [63]. A recent study by Vit et al. claimed DT-061 may not be binding to PP2A; however, a reply published by Leonard et al. refuted many of the assertions made about DT-061 stating a lack of reproducibility of the original studies [64, 65]. In addition, DT-061 was recently assessed in glioblastoma and found to bind with PP2A-B56, bringing further debate into the mechanism of action [66]. Though the SMAPs have been shown to be a promising lead as therapeutics for the direct activation of PP2A in cancer, phenothiazine derivatives are still being developed and assessed for their potential as PP2A activators. Recently, a few novel tricyclic sulfonamides, known as ATUX-3365, ATUX-8385, and ATUX-792, have been synthesized and found capable of activating PP2A in hepatoblastoma and glioblastoma [67, 68]. Furthermore, a phenothiazine derivative, known as iHAP1, was recently thought to induce PP2A activation effectively in T-cell acute lymphoblastic leukemia (T-ALL); however, there have been some contradictory reports related to the anti-neoplastic effect of iHAP1. This raised a question as to whether iHAP1 is working as a PP2A activator or as a tubulin polymerization inhibitor [64, 69]. Though iHAP1 has been observed to inhibit T-ALL, its mechanism of action based on these more recent findings is not clear. Nevertheless, some phenothiazine derivatives have been proven to mechanistically activate PP2A in cancer [55, 57].

Now, there are other direct PP2A activators that are not associated with phenothiazines. While these PP2A activators cannot be put together into a specific class, there are a few compounds of note that have recently been recognized. For instance, we have found NSC49L to be effective against colon cancer through the activation of PP2Ac and subsequently decreasing the AKT1/mTOR/4E-BP1 axis and p21 translation in FOLFOX-resistant colon cancer cells [70]. Moreover, therapeutics traditionally used for the treatment of other diseases have been identified to activate PP2A in cancer. Metformin, an anti-diabetic therapeutic agent, has shown anti-neoplastic activity in lung, prostate, and breast cancers [71, 72]. Through its PP2A activation, metformin has been shown to result in the dephosphorylation of Bax, c-Myc, and AKT1. Further, it has also been attributed with disruption of the α4-PP2Ac complex giving an indication of the mechanism behind the metformin-induced PP2A activation [71, 72]. Sodium selenate, known for its role as an inorganic form of selenium supplementation, was found to activate PP2A in hormone-refractory prostate cancer leading to the downstream inhibition of angiogenesis [73, 74]. Sodium selenate was able to progress into a “first-in-human” phase I clinical trial in castration-resistant prostate cancer patients, where it was well-tolerated and had a similar effectiveness as other single agent anti-angiogenesis therapeutics. As the study was only intended to measure the tolerability of sodium selenate in humans, the small level of efficacy observed led to the conclusion that further studies into its effectiveness as a cancer treatment would require a combination with another chemotherapeutic agent [75]. At the same time, further clinical trials of sodium selenate have been focused on evaluating it in the treatment of neurodegenerative diseases due to the PP2A-induced inhibition of hyperphosphorylated tau [76–78]. Though there has not been much progression of direct PP2A activators into clinical trials for cancer treatment, there are several PP2A modifiers which are being considered for therapeutic development in cancer treatments (Table 1).

### Indirect PP2A activators

The indirect activation of PP2A relies on the inhibition of the endogenous inhibitors of PP2A, such as SET and CIP2A. SET and CIP2A, as previously discussed, are highly expressed and assist in the reduction of PP2A activity in cancer cells [79]. The introduction of a therapeutic intervention can inhibit these endogenous inhibitors by preventing their binding to PP2A (Figs. 1 and 3). Some of the indirect activators have been previously studied in other diseases or been identified for different cancer targets (Table 2) [79]. Displaying the importance that repurposing established therapeutics can have as potential cancer treatments, though novel therapeutics are still being developed as indirect PP2A activators.

**Table 2:** Indirect PP2A activators

Compounds	Cancer Types	Mechanisms	References
FTY720	Acute myeloid leukemia	Impairs interaction of PP2Ac with SET	[81]
	Neuroblastoma	Inhibits proliferation, migration, and invasion	[68, 136]
	Chronic myeloid leukemia	Impairs interaction of SET-PP2A, inhibits the BCR-ABL1 recruitment of JAK2, and activates GSK-3β	[137]
	Colorectal cancer	Decreased phosphorylation of AKT1 and Erk1/2	[79]
	Breast cancer	Increases activation and expression of ERα and inhibits histone deacetylase	[23, 138]
	Oral squamous cell carcinoma	Decreased phosphorylated GSK-3β	[25]
	Multiple myeloma	Ferroptosis, autophagy, apoptosis, and ROS activation	[84, 85]
	Ovarian cancer	Autophagy and non-apoptotic cell death	[88]
	Gastric cancer	Increases PTEN and p53, decreases p-AKT1 and MDM2, induces G1 cell cycle arrest and apoptosis	[87]
	Lung cancer	Decreases S1P-induced IL-6 and IL-8 and TNF-induced COX2, activation of PP2A-RIPK1-dependent necroptosis	[82, 86]
	Hepatocellular carcinoma	ROS-dependent activation of PKCδ and caspase-3-dependent apoptosis	[139]
CM-1231	Acute myeloid leukemia	Targets SET	[91]
OSU-2S	Acute myeloid leukemia	Impairs interaction of SET and PP2Ac, c-Myc degradation and increased p21	[90]
	Chronic lymphocytic leukemia	Nuclear translocation of SHP1^S591^	[89]
OP449	Acute myeloid leukemia	Antagonizes SET	[81, 140]
	Chronic myeloid leukemia		[140]
Bortezomib	Triple negative breast cancer	Inhibits CIP2A, decreased p-AKT1	[78, 79]
	Colorectal cancer		
Ethoxysanguinarine (ESG)	Lung cancer	Inhibits CIP2A, decreased c-Myc and p-AKT1, apoptosis	[94]
TD52	Triple negative breast cancer	Inhibits CIP2A, interrupts ELK1 binding to CIP2A promoter, decreased p-AKT1	[93]
	Hepatocellular carcinoma		[95]
Arctigenin	Triple negative breast cancer	Inhibits CIP2A	[141]
Celastrol	Gastric cancer	CIP2A degradation, increased apoptosis	[142]
Niclosamide	Non-small cell lung cancer	Inhibits CIP2A, increased mitochondrial ROS	[143]
Polyphyllin I	Prostate cancer	Inhibits CIP2A and ERK	[99]
Lapatinib	Triple negative breast cancer	Inhibits CIP2A and p-AKT1	[98]

One of the well-known indirect PP2A activators is FTY720, a sphingosine immunosuppressor approved by the Food & Drug Administration (FDA) for the treatment of relapsed multiple sclerosis (MS) [80]. Yet, FTY20, also known as fingolimod, has been shown to activate PP2A by directly impairing the interaction of PP2Ac with SET [81, 82]. The binding of SET to PP2Ac involves both the amino terminus and the carboxy terminal of SET. As a result, the binding of FTY720 to the last 100 amino acids (177-277AA) of the SET carboxy-terminus thwarts the typical binding of SET to PP2Ac, allowing for the activation of PP2A [81, 82]. In fact, it was discovered that the amino acid K209 is required in the binding of FTY720 to SET, indicating further confirmation of the residues involved [81, 82]. Moreover, it was observed that SET tends to accumulate in the cytoplasm of hematologic malignant cells and treatment with FTY720 results in the accumulation of SET in the nucleus. This results in less SET available to interact with the cytoplasmic PP2A allowing for its increased activation [81, 83]. Through this core mechanism, FTY20 has been shown to inhibit multiple cancer types such as colorectal, breast, ovarian, gastric, lung, oral, neuroblastoma, leukemias, myeloma, and hepatocellular carcinoma [22, 23, 25, 81, 84–88]. For instance, in oral squamous cell carcinoma FTY720 reduced cancer progression through indirect dephosphorylation of GSK-3β and decreased the phosphorylation and activation of PP2Ac at Y307, though use of the p-Y307 antibody in this study likely invalidates this observation [19, 25]. Additionally, the FTY720 treatment in lung cancer resulted in the activation of PP2A-RIPK1-dependent necroptosis, while it decreased S1P-induced IL-6 and IL-8 expression, as well as TNF-induced COX2 expression [82, 86]. Generally, the precise downstream mechanism of FTY720 is dependent on cancer type beyond its primary interaction with SET. Nevertheless, FTY720, despite its broad range of effectiveness in cancer, has some limiting side-effects, such as cardiotoxicity and lymphopenia, a result of T-cell sequestering to the lymph node. Both side-effects occur due to the interaction of FTY720 with the sphingosine-1-phosphate receptor (S1PR), which is the primary mechanism for its use in relapsed multiple sclerosis [89–91]. To address this hindrance, many FTY720 analogues have been developed for cancer therapy [89, 91]. For instance, recently OSU-2S, an analogue of FTY720, has been developed for the treatment of acute myeloid leukemia (AML) and chronic lymphocytic leukemia (CLL) through the inhibition of SET, where it results in the interference of the c-Myc/p21 and SHP1 [89, 90]. Though OSU-2S has not yet been resolved of these side effects, another FTY720 analogue, CM-1231, did not induce any cardiac toxicity in zebrafish and was shown to perform better than FTY720 in the treatment of AML [91]. Overall, FTY720 has shown intriguing results in inducing cancer inhibition through PP2A activation; yet the side-effects of the original compound have limited its use in cancer treatment. However, since many groups are developing analogues of FTY720 to address its failings, there may be a FTY720-like compound capable of further applications in cancer therapy.

Though CIP2A is also one of the known endogenous inhibitors of PP2A, a great deal is still being discovered into the way it functions as an oncogene and prevents the activation of PP2A in cancer [92, 93]. Presently, it is known that CIP2A homodimerizes and interacts with PP2A-B56α and PP2A-B56γ through their conserved N-terminal amino acids; however, the mechanism behind the inhibition of CIP2A seems to be through downregulating its transcription [93, 94]. To give an example, TD52, an erlotinib analogue, was found to prevent the binding of Elk1 to the CIP2A promoter, resulting in decreased CIP2A transcription in hepatocellular carcinoma [95]. Bortezomib was found to cause decreased CIP2A expression in colon cancer, while its use in triple negative breast cancer resulted in reduced *CIP2A* mRNA expression and no variation in CIP2A degradation [96, 97]. Though the core mechanism behind CIP2A inhibitors appears to be through the inhibition of CIP2A transcription rather than its binding to PP2A, the overall effect of increasing PP2A activity in cancer cells is still comparable to the SET inhibitors. This can be seen in the altered effects observed in the proteins in the downstream signaling pathways of PP2A, such as AKT1, c-Myc, and ERK [94, 98, 99].

## PP2A inhibitors

The core objective behind targeted inhibition of PP2A in cancer treatment is linked to the role PP2A plays in DNA damage/repair and cell cycle progression [8]. When DNA damage occurs PP2A will assist in the halt of cell cycle progression and the initiation of DNA damage repair [8]. For example, PP2A is known to dephosphorylate p53 at S37 and T55 leading to increased stability of p53 and the activation of the DNA damage response [100, 101]. It has also been implicated in the dephosphorylation of γ-H2AX, which is required for cells to be released from DNA double stranded break repair [102, 103]. As such, therapeutics causing PP2A inhibition can block this response to DNA damage forcing cancer cells with damaged DNA to undergo cell cycle progression, prevent the release of cells from DNA damage repair, etc. and ultimately cause cytotoxicity due to accumulation of damaged cells and unstable chromatin [8, 102]. The idea of targeting PP2A for either activation or inhibition in the same disease exhibits a vast contradiction that can be explained by the involvement of different regulatory subunits of PP2A in determining the ultimate response to cancer cells. Since PP2A supports the regulation of several signaling pathways involved in cancer progression, therapeutics modifying the activity of PP2A rely on its substrate specificity to target the desired signaling pathways involved in cancer [8, 51].

There are many PP2A inhibitors with observed tumor inhibition activities; however, none of them have progressed into approved clinical use. These PP2A inhibitors have been shown to inhibit cancer progression in numerous cancer types both in vitro and in vivo (Table 3) (Fig. 3). For instance, cantharidin, a toxin from blister beetles, and its demethylated form, noncantharidin, have been shown to inhibit cancers, such as hepatoma, myeloma, and multidrug-resistant leukemia through PP2A inhibition [104–107]. Cantharidin, capable of binding to both PP2A and PP1, has a higher binding affinity for PP2A; nevertheless, the significant toxicity obtained with this compound limits its clinical application [8, 108]. Fostriecin, a selective PP2A and protein phosphatase 4 (PP4) inhibitor, underwent a Phase I clinical trial; however, before the MTD could be established, the study had to be concluded early due to problematic concerns about the purity and stability of the fostriecin supply [109, 110]. Still, fostriecin primarily functions by inhibiting the mitotic entry point in cancer cells, such as leukemia, ovarian cancer, and small-cell lung carcinoma [109–112]. Another common PP2A inhibitor utilized often in cancer research is okadaic acid (OA), a marine toxin obtained from the black sponge *Halichondria okadai* [113]. OA is an inhibitor for PP2A and PP1, but it can also inhibit other protein phosphatases, such as PP4, PP5, and PP2B [113]. Though OA is mostly used as a research tool for its capabilities as a tumor promoter and a PP2A inhibitor, there have been some evaluations into its uses as a therapeutic agent. It has been shown that OA can inhibit cellular proliferation of lung cancer cells alone or in combination with cisplatin [114, 115]. While there are many therapeutic inhibitors for PP2A, most of them are not suitable for further progression towards clinical trials due to concerns of toxicity and stability.

**Table 3:** PP2A inhibitors

Compound	Cancer Types	Mechanisms	References
LB-100	Medulloblastoma	Decreased activation of STAT3 (↑Ser727-p-STAT3)	[144]
	Glioblastoma	Activates Plk-1 and AKT-1, inhibits p53	[118]
	Pancreatic cancer	Activates CDC25C/CDK1, inhibits homologous recombination repair (HRR)	[120]
Cantharidin	Pancreatic Cancer	G2/M cell cycle arrest, decreased CDK1, and increased p21	[107]
	Hepatocellular carcinoma	Binds to EphB4, regulating JAK2/STAT3 pathway	[145]
Noncantharidin	Colorectal cancer	Activates the CD95 receptor/ligand	[146]
Okadaic Acid	Lung cancer	Induces apoptosis	[114, 115]
Calyculin A	Breast cancer	Increases p-Cyclin D1 and initiates its degradation	[147]
Tautomycin	Medullary thyroid cancer	Inhibition of GSK-3β	[148]
Tautomycetin	Medullary thyroid cancer	Inhibition of GSK-3β	[148]
Fostriecin	Leukemia	Binds to Cys269 residue of PP2Ac	[149]
	Small-cell lung carcinoma	Inhibits TopoII without forming a complex with DNA and TopoII	[111]
	Ovarian cancer	Activates STAT1	[112]

Despite many PP2A inhibitors only being used in research due to toxicity concerns apparent in clinical use, further development of these compounds has occurred by optimizing their therapeutic properties in cancer through the design of potent analogues. LB-100 is one such analog, where as a cantharidin analogue, it was designed to achieve a better therapeutic profile in cancer cells than the parent compound [51, 116]. LB-100 has been recognized for its inhibition of many cancer types through PP2A inhibition. Though, it has been found to also be capable of inhibiting PP5, as well as PP2A, due to a similar catalytic mechanism that PP2Ac shares with PP5c [117]. Nevertheless, in glioblastoma, LB-100 has been found to activate Plk-1 and AKT1 and decrease p53 [118]. Additionally, investigations into LB-100 in nasopharyngeal carcinoma observed an increase in p-Plk-1, TCTP, and Cdk1 along with a decrease in p53 [119]. The effect of LB-100 treatment sensitizes pancreatic cancer cells to radiation by activating CDC25C/CDK1 and inhibiting the homologous recombination repair (HRR) pathways, suggesting that LB-100 can activate cell cycle progression and stall DNA damage repair mechanisms in cancer cells through inducing PP2A inhibition [120]. Furthermore, LB-100 has progressed into a Phase I clinical trial to establish its safety, tolerability, and preliminary antitumor activity in patients with solid tumors [121]. During this study, 29 patients were treated with escalating dosages of LB-100 in a treatment cycle of daily intravenous (I.V.) injections for 3 days every 3 weeks. Though 20.7% of patients experienced drug-related grade 3 adverse events (anemia, decreased creatinine clearance, etc.), 50% of patients in this trial attained disease stabilization for 4 or more cycles without limiting toxicity [121]. Through these findings these investigators were able to establish a relatively good safety and tolerability profile of LB-100 in patients, and additionally, there appeared to be some degree of tumor inhibition as well. These findings gave high indications for the likelihood of LB-100 to further advance into a higher phase of clinical trials. As such, LB-100 is currently undergoing three Phase Ib/II, I, and II clinical trials in myelodysplastic syndromes, small cell lung cancer, and glioblastoma, respectively [122–124].

## Combination therapies and applications in drug resistant cancer

The use of combination therapies is more likely to resolve some of the common issues in cancer therapeutic treatment, such as synergistically increasing the effectiveness of monotherapies without requiring an increase in dosage that would likely lead to higher toxicities. Additionally, combination therapies have also been shown to be a solution towards treating drug-resistant cancers [125–127]. In this regard, multiple PP2A therapeutic modulators have been studied as to their ability to function as combination therapies with other known cancer therapeutics (Table 4). At present, only LB-100, a PP2A inhibitor, is undergoing clinical trials as both a monotherapy and as a part of a combination treatment in cancer patients [122–124]. Specifically, LB-100 is undergoing a Phase Ib clinical trial to find the optimal dosage when used in combination with carboplatin, etoposide, and atezolizumab in the treatment of extensive-stage small cell lung cancer. LB-100 is expected to increase cell division through the inhibition of PP2A allowing the other chemotherapeutics to target the dividing cells for cytotoxicity [122]. Currently, there are no PP2A modifiers that have progressed further than clinical trials for cancer treatments, whether as a mono- or a combination therapy. The only known PP2A modifiers that have progressed into clinical trials for cancer treatment are sodium selenate and LB-100, though in the case of sodium selenate it was determined that a combination therapy would be the most optimal use of this therapeutic agent in cancer [75, 121].

**Table 4:** Combination therapy of PP2A modulators

Compound	Cancer Types	Therapeutic Combinations	References
FTY720	Cisplatin-resistant melanoma	Cisplatin, 5ʹFU, Oxaliplatin	[130]
	Acute myeloid leukemia	Venetoclax	[150]
	Erythroleukemia	A-1331852	
	Colorectal cancer	Cisplatin, 5ʹFU, Oxaliplatin, Doxorubicin (DOX), Etoposide (VP16), SN-38	[22, 127]
	Gastric cancer	Cisplatin	[87]
CM-1231	Acute myeloid leukemia	Venetoclax	[150]
NSC49L	Colorectal Cancer	TRAIL	[70, 131]
	FOLFOX-resistant Colorectal Cancer		
LB-100	Cisplatin-resistant ovarian carcinoma	Cisplatin	[128]
	Medulloblastoma		[144]
	Osteosarcoma		[129]
	Glioblastoma	Anti-CAIX CAR-T therapy, Temozolomide, Doxorubicin	[116, 118]
	Pancreatic cancer	Doxorubicin	[151]
	Hepatocellular carcinoma		[152]
	Pheochromocytoma	Temozolomide	[153]
	JAK2-driven myeloproliferative neoplasms	Ruxolitinib	[154]
Forskolin	Acute myeloid leukemia	Idarubicin and Ara-c	[24]
OP449	Acute myeloid leukemia	Imatinib, Nilotinib, Dasatinib	[140]
	Chronic myeloid leukemia		
	Prostate cancer	Enzalutamide	[155]
Bortezomib	Hepatocellular carcinoma	CS-1008	[156]
Okadaic acid (OA)	Chronic myeloid leukemia	Dasatinib	[157]
	Lung cancer	Hematain	[158]
Ethoxysanguinarine (ESG)	Lung cancer	Cisplatin	[94]
SMAPs	TKI-resistant advanced lung adenocarcinoma	TKI Afatinib	[61]
	Acute myeloid leukemia	Venetoclax	[150]
	Breast cancer	Fluphenazine (Flu)	[132]
	Lung cancer		
	Melanoma		
	Pancreatic ductal carcinoma	INK128	[159]
	Heterogenous Glioblastoma	UCN-01, MK-2206	[66]
	Medulloblastoma		
	High-grade serous carcinoma (HGSC)	Olaparib	[133]

In reviewing the effect of PP2A inhibitors in drug-resistant cancers or combination therapy treatments, LB-100 will be the main consideration. While LB-100 does work alone, it predominantly has been shown to work best when in combination with other cancer therapeutics. For instance, LB-100 has been shown to enhance the anti-CAIX CAR-T therapy for glioblastoma in vitro and in vivo through activation of the mTOR pathway [116]. Moreover, LB-100 is capable of sensitizing cisplatin-treated ovarian carcinoma in vitro and in vivo via the DNA-damage response pathway and nullifying the cisplatin induced S-phase cell-cycle arrest, allowing cells to progress through mitosis [128]. Similarly, the effects of combining LB-100 with either temozolomide or doxorubicin, both DNA disrupting agents, in treating glioblastoma multiforme xenografts resulted in tumor regression [118]. In osteosarcoma, LB-100 and cisplatin treatments resulted in decreased ATM/ATR-mediated DNA damage response, leading to hyperphosphorylation of checkpoint kinase 1 (Chk1) and Chk2, and subsequently causing an increase of cyclin activity in these cells [129]. Essentially, these studies were able to demonstrate that the PP2A inhibitor combined with a DNA disrupting agent will result in DNA-damaged cancer cells progressing through the cell cycle, ultimately leading to cell death via mitotic catastrophe [118, 128, 129].

There are multiple PP2A activators proven to work synergistically when in combination with other cancer therapeutics in drug-sensitive and -resistant cancer cells. One such activator is FTY720, an indirect PP2A activator, which was able to work in combination with cisplatin to induce cytotoxicity in cisplatin-resistant melanoma, gastric cancer, and colon cancer. FTY720 synergistically induced cytotoxicity in colon cancer cells with 5-fluorouracil (5-FU), oxaliplatin, doxorubicin (DOX), and etoposide (VP16) [79, 87, 127, 130]. FTY720 assisted in the VP16-induced apoptosis due to a prolonged accumulation of intracellular DOX in both 5-FU-sensitive and -resistant colon cancer cells, where it also inhibited P-glycoprotein and the multidrug resistance protein 1 (MRP1), the higher level of which are fundamental towards the drug resistance in cancer cells [127]. In recent studies, we found that the direct PP2A activator, NSC49L, has a synergistic cytotoxicity in FOLFOX-resistant colon cancer cells both by itself and in combination with TRAIL, where the NSC49L-induced downregulation of p21 translation allowed the TRAIL-mediated increase in apoptosis [70, 131]. Other studies showed that fluphenazine (Flu), a phenothiazine derivative known to inhibit calmodulin, was able to work with DT-061 in breast, lung, and melanoma cancer cells through the activation of PP2A and inhibition of calmodulin [132]. DT-061 was also found to sensitize high-grade serous carcinoma (HGSC) with homologous recombination (HR) proficiency to the PARP inhibitor, Olaparib, by decreasing RAD51 protein expression. The loss of RAD51, which is essential to the HR pathway for repair of DNA double-stranded breaks, leads to increased DNA-damage in HGSC cells resistant to PARP inhibition [133]. Recently, a triple combination therapy was discovered for the treatment of heterogenous glioblastoma and medulloblastoma, where it was effective in vitro and in vivo with intracranial models. DT-061 combined with the AKT inhibitor, MK-2206, and the pyruvate dehydrogenase kinase (PDK) inhibitor, UCN-01, resulted in a synergistic cytotoxic effect. As the AKT and PDK inhibitors treated together were only able to produce a cytostatic effect, the addition of a PP2A activator caused the induction of apoptosis, inhibition of mitochondrial oxidative phosphorylation, and increased mitochondrial proton leakage [66]. There are multiple combinations of PP2A activators with known cancer therapeutics proven to be effective in inhibiting cancer cell growth in both drug-sensitive and drug-resistant cancers and more are still being discovered.

## Conclusions

The modulation of PP2A activity has verifiably been shown to have an overall effect on the progression of cancer. This is seen in the deregulation of PP2A activity in cancer patients as well as the tumor promoting capabilities of PP2A inhibitors, such as OA. This can also be seen in the therapeutic modulation of PP2A activity leading to cancer inhibition demonstrating the important role PP2A has in cancer. There have been many PP2A modulators developed in the past, yet none of them have progressed towards active clinical application as cancer therapy. Still, sodium selenate has a better tolerability in prostate cancer patients, FTY720 has already been approved by the FDA for clinical use in MS, and LB-100 is proceeding through Phase II clinical trials. All of these are displaying the growing possibilities for the future use of PP2A modulators in the treatment of cancer; however, there may be more use seen of these modulators as combination treatments. As the PP2A therapeutics have been able to sensitize cancer cells to well-known chemotherapeutics or radiation therapy, it brings these compounds into further significance, especially in the case of drug-resistant cancer, which requires more avenues of addressing ways to sensitize these cancers with PP2A modulators being one such possibility.

Regarding the future possibilities of PP2A modulators as cancer therapeutics, it has been highly suggested that achieving substrate specificity by directly targeting specific PP2A regulatory complexes would uphold better tolerability and cancer inhibition. This substrate specificity has been observed to occur in some of the direct PP2A activators, whereas at this point similar findings for the indirect activators or inhibitors have not been observed. For instance, the phenothiazine derivatives, SMAPs, or more specifically DT-061 has shown remarkable target specificity by binding exclusively with the PP2A-B56α complex [55, 57, 61]. The specificity of direct PP2A activation may lead to better options for combination therapy treatments in being able to dual target tumorigenic pathways. Though LB-100 has been successful up to this point in clinical trials, there have been some findings indicating that it does not selectively induce PP2A inhibition. Instead, it might dually inhibit PP2A along with PP5 where it has been proposed that this could be a synergistic mechanism for the observed tumor inhibition of LB-100 [117]. Still, LB-100 is showing significant results as a cancer treatment both as a mono- and combination therapy demonstrating the value that PP2A modulators can bring as cancer therapeutics.

Currently, there are still challenges to overcome in the development of a PP2A modulator for clinical use. Many of the discussed PP2A therapeutics were revealed to have concerns in the form of adverse effects, toxicity, stability, or selectivity issues hindering their progress as clinically relevant therapeutics. Nevertheless, the preclinical findings of these compounds indicate that targeting PP2A in cancer has a strong potential for clinical use. Further optimization of the existing compounds, using rational drug design, and having better understanding of the target protein are some of the strategies applied to use the opportunity provided by these underdeveloped PP2A modulators. Moreover, modern techniques such as AI may even play a role in the future development of PP2A modulators. This is evident when evaluating the lessened adverse effects and increased effectiveness seen with analogs developed from these original PP2A therapeutics, though further optimization is likely still needed for progression into clinical practice. Development of more effective, selective, and clinically relevant small molecule PP2A activators may thus provide a promising option for cancer therapy in the future.

## Data Availability

Not applicable.
